# Whole-Blood RNA Profiles Associated with Pulmonary Arterial Hypertension and Clinical Outcome

**DOI:** 10.1164/rccm.202003-0510OC

**Published:** 2020-08-15

**Authors:** Christopher J. Rhodes, Pablo Otero-Núñez, John Wharton, Emilia M. Swietlik, Sokratis Kariotis, Lars Harbaum, Mark J. Dunning, Jason M. Elinoff, Niamh Errington, A. A. Roger Thompson, James Iremonger, J. Gerry Coghlan, Paul A. Corris, Luke S. Howard, David G. Kiely, Colin Church, Joanna Pepke-Zaba, Mark Toshner, Stephen J. Wort, Ankit A. Desai, Marc Humbert, William C. Nichols, Laura Southgate, David-Alexandre Trégouët, Richard C. Trembath, Inga Prokopenko, Stefan Gräf, Nicholas W. Morrell, Dennis Wang, Allan Lawrie

**Affiliations:** ^1^ National Heart and Lung Institute, Imperial College London, London, United Kingdom; ^2^ Department of Medicine, University of Cambridge, Cambridge, United Kingdom; ^3^ Sheffield Institute for Translational Neuroscience; ^4^ Department of Infection, Immunity & Cardiovascular Disease, and; ^5^ Sheffield Bioinformatics Core, The University of Sheffield, Sheffield, United Kingdom; ^6^ Critical Care Medicine Department, NIH Clinical Center, Bethesda, Maryland; ^7^ University College London, London, United Kingdom; ^8^ Newcastle University, Newcastle upon Tyne, United Kingdom; ^9^ University of Glasgow, Glasgow, United Kingdom; ^10^ Royal Papworth Hospital, Cambridge, United Kingdom; ^11^ Indiana University, Indianapolis, Indiana; ^12^ Université Paris-Sud, Faculté de Médecine, Université Paris-Saclay, Le Kremlin-Bicêtre, France; ^13^ Service de Pneumologie, Hôpital Bicêtre, Assistance Publique – Hôpitaux de Paris, Le Kremlin-Bicêtre, France; ^14^ INSERM UMR_S 999, Hôpital Marie Lannelongue, Le Plessis Robinson, France; ^15^ Division of Human Genetics, Department of Pediatrics, Cincinnati Children’s Hospital Medical Center, University of Cincinnati College of Medicine, Cincinnati, Ohio; ^16^ Molecular and Clinical Sciences Research Institute, St. George’s University of London, London, United Kingdom; ^17^ INSERM UMR_S 1219, Bordeaux Population Health Research Center, University of Bordeaux, Bordeaux, France; ^18^ Division of Genetics and Molecular Medicine, King’s College London, London, United Kingdom; ^19^ Department of Clinical and Experimental Medicine, University of Surrey, Guildford, United Kingdom; and; ^20^ NIHR BioResource for Translational Research, Cambridge, United Kingdom

**Keywords:** pulmonary arterial hypertension, RNAseq, whole-blood RNA

## Abstract

**Rationale:** Idiopathic and heritable pulmonary arterial hypertension (PAH) are rare but comprise a genetically heterogeneous patient group. RNA sequencing linked to the underlying genetic architecture can be used to better understand the underlying pathology by identifying key signaling pathways and stratify patients more robustly according to clinical risk.

**Objectives:** To use a three-stage design of RNA discovery, RNA validation and model construction, and model validation to define a set of PAH-associated RNAs and a single summarizing RNA model score. To define genes most likely to be involved in disease development, we performed Mendelian randomization (MR) analysis.

**Methods:** RNA sequencing was performed on whole-blood samples from 359 patients with idiopathic, heritable, and drug-induced PAH and 72 age- and sex-matched healthy volunteers. The score was evaluated against disease severity markers including survival analysis using all-cause mortality from diagnosis. MR used known expression quantitative trait loci and summary statistics from a PAH genome-wide association study.

**Measurements and Main Results:** We identified 507 genes with differential RNA expression in patients with PAH compared with control subjects. A model of 25 RNAs distinguished PAH with 87% accuracy (area under the curve 95% confidence interval: 0.791–0.945) in model validation. The RNA model score was associated with disease severity and long-term survival (*P* = 4.66 × 10^−6^) in PAH. MR detected an association between SMAD5 levels and PAH disease susceptibility (odds ratio, 0.317; 95% confidence interval, 0.129–0.776; *P* = 0.012).

**Conclusions:** A whole-blood RNA signature of PAH, which includes RNAs relevant to disease pathogenesis, associates with disease severity and identifies patients with poor clinical outcomes. Genetic variants associated with lower SMAD5 expression may increase susceptibility to PAH.

**At a Glance Commentary**

**Scientific Knowledge on the Subject**

Blood transcriptomic profiles in pulmonary arterial hypertension have been examined in small limited studies indicating their potential utility in differentiating patients from control subjects and by clinical phenotypes.

**What This Study Adds to the Field**

This study represents the most comprehensive analysis of whole-blood RNA profiles in pulmonary arterial hypertension and identifies and validates a signature that both differentiates patients from control subjects and stratifies patients by clinical risk and severity. Genetic analyses (Mendelian randomization) indicate potential causal roles for some genes including the TGF-β signaling molecule SMAD5.

Pulmonary arterial hypertension (PAH) is associated with vasoconstriction and occlusion of distal pulmonary arteries, characterized by endothelial damage, smooth muscle and fibroblast proliferation, and inflammation. Increased pulmonary vascular resistance leads to right heart failure, with survival rates estimated at 52–75% at 5 years, even with modern-day therapy (1). The rate of deterioration and response to therapy varies between patients, driving a search for better predictors of clinical outcome and tools to inform drug selection. Molecular profiling using multiple omics technologies offers greater granularity than standard clinical phenotypes for characterizing patients with PAH and could improve initial risk stratification, treatment selection, and monitoring as well as provide insights into biological pathways not yet targeted by current therapies (2–4).

Transcriptome profiling through RNA sequencing permits a comprehensive analysis of gene expression in tissue samples. Whole-blood RNA analysis offers an alternative “liquid biopsy” to lung biopsy, which carries a high risk in PAH, and can be performed sequentially. This approach can also investigate immune mechanisms in PAH that have recently been highlighted (5). Previous studies of blood RNA in PAH have been limited by patient numbers and the use of microarrays, which are less sensitive than high-quality RNA sequencing (RNAseq) and limited by the probe set of each specific array. A recent meta-analysis of these microarray studies identified some consistent differentially expressed genes not appreciated in individual studies (6). Explanted lung tissues from patients with late-stage PAH have also shown differences in RNA profiles (7). The results from both studies remain to be validated in independent cohorts.

The aim of this study was to characterize gene pathways associated with PAH and to assess their association with disease heterogeneity, specifically in terms of disease severity and outcomes including response to vasodilators and mortality, and genetic background. We compare gene expression in whole-blood samples from 359 patients with idiopathic, heritable, or drug-induced PAH from the UK PAH Cohort study with 72 age- and sex-matched healthy volunteers without any cardiac or respiratory disease as control subjects. Using equal distribution of samples into a three-stage design, we identified reproducible RNA expression differences by RNAseq. Two distinct computational approaches were used to estimate white blood cell (WBC) fractions and thus account for the potential effect of different cell numbers on gene transcript levels across samples. A predictive statistical model that combined gene expression differences performed well at identifying patients with PAH in a separate case–control analysis. The RNA-based score was also associated with disease severity and clinical outcomes (all-cause mortality). Enrichment of genetic variants that determine the levels of PAH RNAs was detected, implicating these RNAs in the pathogenesis of the disease.

## Methods

Comprehensive methods are included in the online supplement.

### Study Participants and Sample Analysis

Patients with idiopathic, heritable, or drug-induced pulmonary arterial hypertension (referred to throughout as PAH) were recruited from expert centers across the UK as part of the PAH Cohort study (www.ipahcohort.com). In each case, diagnosis was confirmed by right heart catheterization following established international guidelines (1), which remained unchanged for the duration of this study. Healthy volunteers were recruited at the same centers and samples processed using the same standard operating procedure at all sites. All individuals gave written, informed consent with local ethical committee approval. Whole blood (3 ml) was collected in Tempus Blood RNA Tubes, and RNAseq was performed using established Illumina methodologies (*see* online supplement for further details). Genomics data were obtained from a published PAH genome-wide association study (8).

Three hundred fifty-nine patients with PAH were randomized into three data analysis groups for RNA discovery (*n* = 120), RNA validation (*n* = 120), and model validation (*n* = 119). Each of these three groups was then compared with an independent set of age- and sex-matched healthy volunteers as control subjects (*n* = 24 in each set; Table 1 and Figure E1 in the online supplement).

**Table 1.** Basic Demographics of Control Subjects and Patients with PAH in Three Analysis Groups and More Detailed Clinical Characteristics, Including Disease Severity of Patients with PAH as a Cohort

	A: RNA Discovery		B: RNA Validation		C: Model Validation	
	Control Subjects	PAH	Control Subjects	PAH	Control Subjects	PAH
Female	17	89	16	80	17	86
Male	7	31	8	40	7	33
Age, yr	43.9 (30.2–53.4)	45.2 (35.7–55)	45.1 (31–53.5)	43.2 (33.3–54.8)	44.4 (31.7–51.6)	47.1 (36.7–61.2)

**PAH Patient Characteristics in A, B, and C**				**Median (25th–75th Percentile) or Counts**		
Age at diagnosis				44.8 (34.3–58.1)		
Female/male				255/104		
Ethnicity, white/other				314/45		
WHO functional class, I/II/III/IV				36/138/155/18		
Six-minute-walk distance, m				330 (224.5–411)		
Mean pulmonary artery pressure, mm Hg				53 (46–61)		
Mean right atrial pressure, mm Hg				8 (6–12)		
Pulmonary capillary wedge pressure, mm Hg				10 (7–12)		
Q̇, L/min				3.8 (3.05–4.9)		
Cardiac index, L/min/m^2^				2.07 (1.68–2.57)		
Pulmonary vascular resistance, Wood units				11.55 (7.93–15.78)		
Years since diagnosis sampled				3.95 (1.38–7.7)		
Years survived since sampling				3.14 (2.3–3.67)		
Years survived since diagnosis				6.99 (4.19–10.88)		
Vasoresponders				21		

*Definition of abbreviations*: PAH = pulmonary arterial hypertension; WHO = World Health Organization.

Control subjects are healthy volunteers without any cardiac or respiratory disease.

### RNAseq Data Analysis

Fastq files (raw reads from RNAseq) were analyzed using Salmon v0.9.1 (9) and GENCODE release 28 to produce transcript abundance estimates that were converted to gene expression data using tximport in *R* with Rstudio (10). Quality control is detailed in the online supplement.

RNAseq analysis of tissues with mixed cell types such as blood can be affected significantly by the cell composition of each sample. Two different computational approaches, CIBERsort (11) and quanTIseq (12), were used to predict WBC profiles associated with PAH, which were included as covariates in secondary differential gene expression analyses (*see* online supplement). Differential expression analysis was performed using edgeR v3.22.5 (13) correcting for the three principal components, which each explained more than 1% of the variance in the data set. Differentially expressed genes were defined in analyses both with and without WBC fractions as covariates in distinct discovery and validation sample sets. These sets were then combined, and only significant genes (*P* < 0.05) directionally consistent in the initial analyses and meeting false discovery rate (FDR) multiple test corrections (based on all detected genes, α = 0.1) in the combined analysis were taken forward. Five hundred seven genes meeting these criteria were considered to generate a model to distinguish PAH from control subjects. Subset selection of RNAs that best distinguish PAH in combination was performed by least absolute shrinkage and selection operator (LASSO) regression analysis, using the glmnet v2.0-18 package from CRAN, with k-fold cross-validation (*k* = 10) selecting the largest value of lambda such that error is within 1 SE of the minimum. This produces an RNA score from a linear weighted combination of the mRNAs identified by the LASSO analysis. Receiver operating characteristic analysis was performed using the pROC v1.14.0 package from Bioconductor (14).

Survival curves from date of diagnostic right heart catheterization were constructed using Kaplan-Meier estimates with left truncation for date of sampling for this study to correct for survival bias. Differences in survival estimates were assessed by log-rank test. RNA scores were also compared across disease severity markers 6-minute-walk test (Spearman’s rank) and World Health Organization (WHO) functional class (Kruskal-Wallis ANOVA) and by cardiac biomarkers (circulating BNP [brain natriuretic peptide] or NT-proBNP [N-terminal pro-brain natriuretic peptide] as available, using cutoffs from European guidelines for risk assessment (1)).

Functional annotation and enrichment of the genes associated with PAH was performed using DAVID (david.ncifcrf.gov) and Ingenuity Pathway Analysis (IPA) using in-built FDR corrections for multiple tests.

Mendelian randomization (MR) analysis using all independent genome-wide significant whole-blood expression quantitative trait loci (eQTL) from two published studies (15, 16) and PAH association from our published genome-wide association study (8) was performed using the TwoSampleMR package (17).

## Results

### Identification of RNAs Differentially Expressed in Patients with PAH Compared with Control Subjects

**Figure 1.**
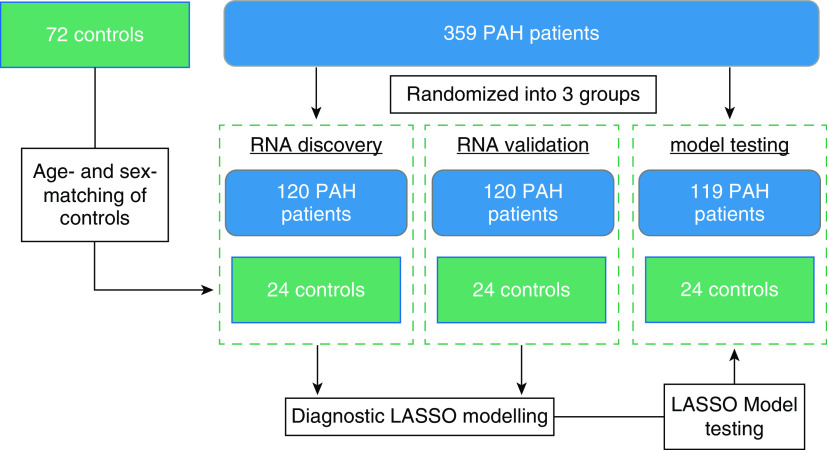
Study design. Three hundred fifty-nine consecutively recruited patients with pulmonary arterial hypertension (PAH) from the UK National Cohort study of PAH were randomized into three groups. Age- and sex-matched control subjects for each group were then identified from the same study. The first two groups were analyzed separately for RNA discovery and RNA validation and then combined for modeling of the best combination of RNAs to distinguish between control subjects and PAH. This model was then tested in the final group of individuals. All patients were combined for subsequent clinical analyses. LASSO = least absolute shrinkage and selection operator.

**Figure 2.**
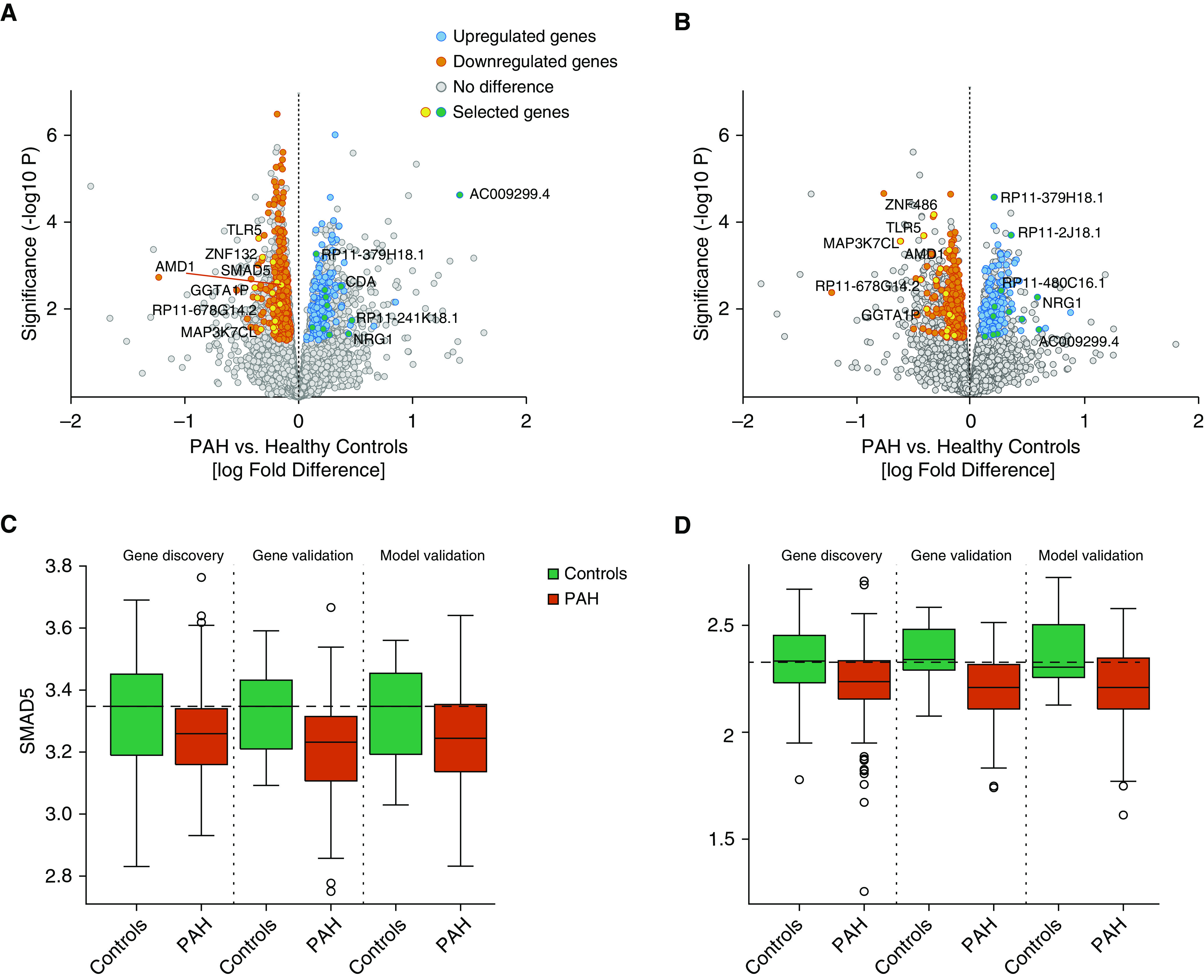
Identification of RNAs with different levels in patients with pulmonary arterial hypertension (PAH) and control subjects. (*A* and *B*) Volcano plot of RNA log fold differences between PAH and control subjects in discovery (*A*) and validation (*B*) analyses. Selected genes were identified in further analyses presented throughout the manuscript including modeling, external validation, and Mendelian randomization. (*C* and *D*) Box plots of SMAD5 (*C*) and TRPC1 (*D*) levels (log_10_ reads) in control subjects and patients with PAH in the RNA discovery, validation, and model validation (validation 2) analysis groups. Horizontal dashed lines in *C* and *D* indicate the median level in healthy control subjects.

The three-stage study design is depicted in Figure 1. Five hundred seven RNAs were significantly different (*P* < 0.05) between PAH and control subjects with directional concordance in both discovery and validation analyses (Figure 2 and Table E3) before and after accounting for WBC fractions (*see* Methods and Tables E1–E3 and Figure E2). All 507 genes were included after accounting for multiple testing (FDR α < 0.1, Table E3). None were associated with exposure to the main PAH therapies (*see* online supplement for further details). These included RNAs in pathways relevant to PAH; for example, *SMAD5* (mothers against decapentaplegic homolog-5), encoding a downstream mediator of signaling of *BMPR2* (3), was reduced in patients with PAH (Figure 2C), consistent with documented reduced BMPR2 signaling in this condition, and the transient receptor potential cation channel, *TRPC1* (Figure 2D), also associated with the development of PH (18), was also reduced in patients.

A set of differentially expressed RNAs relevant to PAH pathobiology were selected for validation of RNAseq quantification by real-time quantitative PCR, namely, *NRG1*, *TRPC1*, *FBLN2*, *SESN1*, *SMAD5*, and *CCND3*. *SPAST* was also selected for its stability across samples as a potential control gene (*see* Methods for details). All real-time quantitative PCR measurements correlated significantly with RNAseq quantification (*P* < 0.05, Spearman’s rho = 0.3–0.89, Table E4).

**Figure 3.**
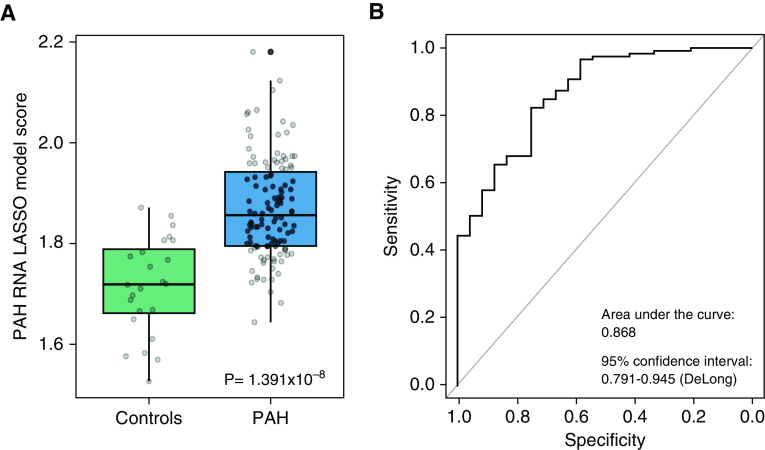
Least absolute shrinkage and selection operator (LASSO) model performance in an independent validation group of age- and sex-matched subjects. (*A*) Box plot showing LASSO model scores for control subjects (*n* = 24) and patients with pulmonary arterial hypertension (PAH) (*n* = 119). (*B*) Receiver operating curve showing the performance of LASSO model scores for determining PAH status in the model validation group.

Patients and control subjects from the RNA discovery and validation analyses were then combined and LASSO analysis was used to determine the combination of RNAs that performed best in discriminating patients with PAH from control subjects in a single model; this analysis yielded 25 RNAs (Table E5). The model was then tested in an independent validation set (*n* = 119 patients with PAH and *n* = 24 control subjects) and demonstrated an area under the curve of 0.868 (95% confidence interval, 0.791–0.945) (Figure 3). The optimum cutoff for identifying PAH with the LASSO model of 1.768 recognized 88.9% of patients with 72.2% specificity.

### Survival Association of RNA Model in Clinically Diagnosed Idiopathic and Heritable PAH

**Figure 4.**
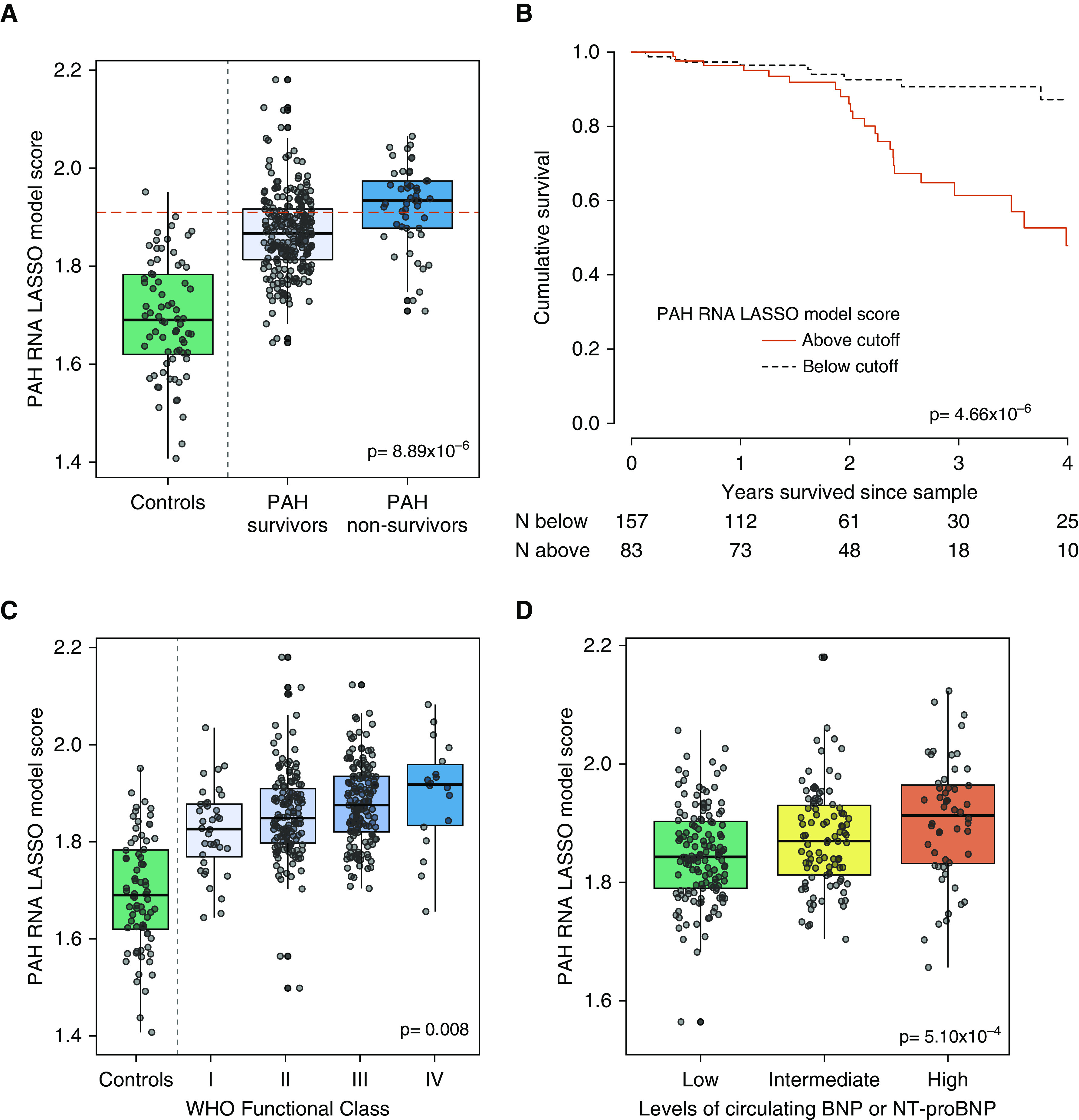
Diagnostic RNA model and survival in pulmonary arterial hypertension (PAH). (*A*) Box plot of LASSO model score in control subjects and patients with PAH separated by survival status during follow-up. Dashed line shows the cutoff that identified 88.9% of nonsurviving patients with PAH. (*B*) Kaplan-Meier survival plot separating patients on the basis of the 88.9% sensitive cutoff. (*C*) Box plot of RNA model score in healthy volunteers and patients with PAH divided by WHO functional class. (*D*) Box plot of RNA model score in patients with PAH divided by presence of low, intermediate, or high levels of cardiac biomarkers BNP (brain natriuretic peptide) (<50, 50–300, or >300 pg/ml) or NT-proBNP (N-terminal pro-brain natriuretic peptide) (<300, 300–1,400, or >1,400 pg/ml), as per European guidelines for PAH assessment. LASSO = least absolute shrinkage and selection operator; WHO = World Health Organization.

We next examined whether the RNA model was also associated with patients with the poorest outcomes, using all 359 patients. The optimum cutoff (1.910) for identifying PAH nonsurvivors with the LASSO model separated patients with PAH into low- and high-risk groups in survival analysis (Figures 4A and 4B and E3).

To determine which of the 25 transcripts in the model were responsible for the association with survival, we tested each transcript for associations with all-cause mortality during follow-up. After FDR correction for multiple testing, three intronic long noncoding RNAs (RP4–751H13.6/ATP6V0E2-AS1, RP4–534N18.4/AL136115.3/Lnc-PTP4A2–13, RP11–701H24.5/lnc-SNRPN-1:6) were associated with survival in all the patients with PAH analyzed and cutoffs distinguished high- and low-risk patient subgroups (Table E6 and Figure E4).

To further characterize the RNA signature, we analyzed its association with three clinical measures of disease severity—WHO functional class, exercise capacity (6-minute-walk), and cardiac biomarkers (BNP or NT-proBNP). We found a significant difference in RNA scores between patients in different WHO functional classes (*P* = 0.008; Figure 4C) and BNP and NT-proBNP levels (*P* = 5.10 × 10^−4^; Figure 4D) and a significant negative correlation with exercise capacity (Spearman’s rho = −0.256; *P* = 8.7 × 10^−5^).

### RNA Profiles in Responders to Vasodilator Therapy

Several RNAs have been proposed as biomarkers for identifying vasoresponders to calcium antagonists using cultured lymphocytes (19). We compared the transcriptome of the 17 vasoresponders and 223 nonvasoresponders in the RNA discovery and first validation groups but found that no transcripts consistently distinguished responders and nonresponders and that none of the previously implicated RNAs were even nominally different (Table E7).

### Comparison with Differentially Expressed Genes Identified in Previous Studies

A meta-analysis of previous transcriptomic studies in blood samples from patients with PAH (6) and a study of PAH lung tissue samples (7) were compared with results from the current RNAseq analysis.

Four hundred sixteen out of the 507 top dysregulated genes from the current RNAseq study were present in the PAH blood transcriptomic meta-analysis (6). Out of those, 300/416 (72%) were directionally consistent with results from the current RNAseq study. One hundred twenty-six out of 300 (42%) of these were nominally significant and 70 met FDR corrected significance. Thirty-seven out of 70 genes also met FDR corrected significance in the idiopathic PAH subgroup analysis from that same study (Table E8).

Three hundred seventy-two out of the 507 top dysregulated genes from the current RNAseq study were present in the lung tissue microarray study and 161/372 (43%) were also directionally consistent. Forty-one out of 161 (25%) genes were nominally significant and 26 met FDR corrected significance (Table E9). Only one gene was found to be dysregulated in patients with PAH across all three studies: *AMD1* (encoding a polyamine biosynthesis intermediate enzyme, adenosylmethionine decarboxylase 1) was consistently lower in PAH.

### Functional Characterization of RNAs Related to PAH

Of the 507 RNAs found to be differentially expressed in PAH, 435 were present in the functional annotations in DAVID. Enrichment of DNA-binding TFs (transcription factors), such as HIF1α (hypoxia-inducible factor 1α) and KLF10 (Krüppel-like factor 10), and many zinc finger–containing TFs was observed compared with a background of the genes detected (Table E10). The Ingenuity Knowledge Base mapped 505/507 transcripts. Double-stranded DNA repair, T-cell receptor, PI3K signaling in B lymphocytes, the role of JAK family kinases in IL6-type cytokine signaling and hypoxia signaling were among the top canonical pathways identified by IPA (Figure E5 and Table E11). *AMD1*, the only gene in common between the current RNAseq study, the recent lung tissue microarray study and the gene expression meta-analysis, was part of the top IPA gene network (Figure E6).

### MR Analysis for Association of RNAs with PAH Development

To determine which of the 507 RNAs associated with PAH are most likely to be causal in disease pathogenesis, we performed a two-sample MR analysis using whole-blood eQTL from two population-based studies (15, 16) and summary statistics from a published PAH genome-wide association study of 2,085 patients with PAH and 9,659 control subjects (8). MR analysis determines whether genetic variation associated with a trait (e.g., high or low RNA expression) is itself associated with a phenotype, in this case the development of PAH. In this exploratory analysis in which eQTL were available for 293/507 RNAs, two genes, *SESN1* (Sestrin-1) and *SMAD5*, reached nominal significance using eQTL in both data sets (15, 16); eleven more reached nominal significance using eQTL from one or the other of the two studies (Tables E12 and E13).

**Figure 5.**
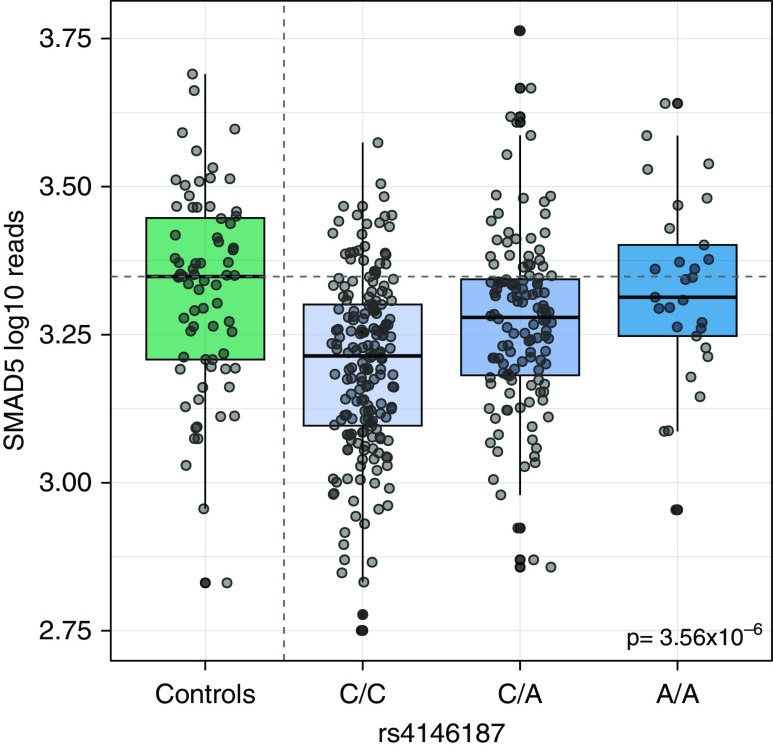
Whole-blood RNA levels of SMAD5 in control subjects and patients with pulmonary arterial hypertension stratified by genotype at the SMAD5 expression quantitative trait locus rs4146187.

The *SMAD5* eQTL SNP rs4146187 was clearly associated with *SMAD5* RNA levels in patients with PAH in this study (*P* = 3.56 × 10^−6^, Figure 5; gnomAD database allele frequency in non-Finnish European population = 0.275). Patients with the A/A genotype had comparable *SMAD5* RNA levels to control subjects, whereas patients with the C/C genotype had a median 18% lower *SMAD5* RNA level. The C/C genotype was present in 49.4% of patients with PAH, and in the PAH genome-wide association study (8), each copy of the A allele (associated with higher *SMAD5* levels) was associated with an 8.5% reduction in the risk of developing PAH (odds ratio, 0.915; 95% confidence interval, 0.846–0.990; *P* = 0.0266). *SMAD5* levels were similarly reduced in patients with PAH with and without pathogenic *BMPR2* variants (Figure E7A), supporting the observation that impaired signaling in the BMPR2 pathway is more common in PAH than rare mutations in *BMPR2* suggest. The PAH RNA model score was similarly elevated in patients with PAH with and without pathogenic *BMPR2* variants (Figure E7B).

## Discussion

Here we report an RNA signature that separates idiopathic and heritable PAH from healthy individuals. The signature also stratifies patients according to disease severity and risk of early death, adding plausibility to its association with PAH. Several of the discriminating mRNAs encode TFs, including SMAD5, HIF-1α and KLF10. MR analysis to integrate genomic data and identify underlying pathogenic signaling pathways revealed that genetic variants associated with lower expression levels of *SMAD5* are more common in patients with PAH.

MR is a powerful tool for separating cause from consequence, as an individual’s genetic status predates the development of PAH. We harnessed genetic data from a published international genome-wide association study (8) and information on previously identified eQTL, common genetic variants that alter gene expression levels, from large published studies in whole-blood RNA samples (15, 16). The identification of SMAD5 by this unbiased strategy has biological plausibility as *SMAD5* encodes an intracellular transcriptional modulator that is activated by ligand binding of BMPR2, the most common genetic risk factor in heritable PAH (3). SMAD5 also has BMP-independent roles, including regulation of cellular metabolism, directly interacting with hexokinase to increase glycolysis, and responding to changes in intracellular pH (20). SMAD5 also controls levels of the master iron regulator hepcidin (21), which may be elevated and drive iron deficiency associated with poor outcomes in PAH (22). Novel therapeutics under development target restoration of BMPR2 signaling in a variety of ways, including ligand or coactivator replacement and allowing read-through of premature stop codons (23–25). Whole-blood SMAD5 profiling may represent an accessible measurement of BMPR2 pathway dysfunction in patients with PAH.

We were able to externally validate our findings with a meta-analysis of published PAH blood transcriptome studies (6) and a PAH lung RNA profiling study (7). The concordance of our whole-blood RNAseq findings is better with blood transcriptomics than with lung tissue data, as might be expected. *AMD1* was the only gene consistently dysregulated in both these studies and our data set, with lower levels in PAH samples in all three studies. *AMD1* encodes a key enzyme controlling the supply of decarboxylated S-adenosylmethionine for polyamine biosynthesis and is regulated by several mechanisms, including increased protein degradation in the presence of elevated polyamines and the inhibition of mRNA translation by spermidine and spermine (26). We have previously demonstrated that elevated circulating levels of polyamine metabolites, such as acisoga, 4-acetamidobutanoate, and N-acetylputrescine, are associated with poor outcomes in PAH (4). Reduced *AMD1* expression in patients with PAH may be in part due to negative feedback from elevated polyamines. These data contrast with observations in hypoxic rodents. *Amd1* expression is increased in hypoxic animals, and both AMD1^+/−^ mice and mice treated with the AMD1 inhibitor, SAM486a, were partially protected from the development of hypoxic PH (27). Interestingly, TRPC1 transcript levels were also reduced in PAH, in contrast to findings in hypoxic mice (18). This might reflect the limitations of the hypoxic rodent as a model of human PAH. Support for further investigation of polyamine metabolism in PAH comes from the recent association of rare deleterious mutations in *ATP13A3* with PAH (3). ATP13A3 is linked with polyamine biosynthesis and is a potential target for drug development (28).

Many of the transcripts that passed robust statistical evaluation in this study are novel. We identified three specific long noncoding RNAs that are reduced in PAH and associated with poor outcomes. These transcripts are not well studied, and their role in regulating lysosomal proton pump protein ATP6V0E2, protein tyrosine phosphatase PTP4A2, or small nuclear ribonucleoprotein-associated protein N remain to be established. Their association with survival selects these out as worthy of further investigation. In addition to suggesting biological relevance, the association of the RNA model, developed to distinguish PAH from control subjects, with survival and disease severity in patients with PAH suggests that the differences observed could be useful to identify patients who may require a more aggressive treatment strategy, such as upfront triple therapy (29). This strategy could also be used to prioritize patients more likely to have events to power clinical trials. RNA profiles and, in particular, RNAs with known eQTLs, could be of further use in clinical trial design to identify patients more likely to respond to specific therapeutics. It would be of great interest, for example, to observe whether patients with lower SMAD5 levels, or simply with the variant associated with lower SMAD5 levels, showed a differential response to novel therapeutics targeting relevant signaling, such as the TGF-β ligand trap, Sotatercept.

There is considerable interest in developing a biochemical test to identify patients with PAH who respond well to calcium antagonists that would supersede the current test, namely, an acute vasodilator challenge while undergoing cardiac catheterization. In contrast to previous reports, we were unable to demonstrate the association of a peripheral transcript signature to vasoresponder status, including previously studied RNAs (19). A strength of our study is the number of patients studied. Previous reports have relied on much smaller patient numbers. That we were able to demonstrate consistency between our main analysis results and those of a recently published meta-analysis of PAH blood transcriptome studies (6) suggests our methodological approaches are robust.

One of the limitations of sampling whole blood to derive transcripts is that samples comprise a mixed population of cells. We used established deconvolution methods to correct for potential confounding in RNA expression analysis. In support of the validity of this approach, we noted altered numbers of regulatory T cells and CD8^+^ T cells in patients with PAH, consistent with previous reports (30, 31), although there is debate on precise changes in cell subpopulations and this may be due in part to differing methodologies and definitions of cell types (32).

This study does not assess the role of post-translational modifications in the pathogenesis of PAH, which could add further information to the circulating transcriptome. It is important that further mechanistic studies consider the role of the genes highlighted in this study in the tissues of primary interest, namely, the lung and heart. The study design included more patient samples than control subjects to account for the higher heterogeneity typically observed in patient populations. This heterogeneity is observed in the overlap in box plots of individual RNA levels or scores between subsets of patients with PAH and control subjects and emphasizes the importance of using any molecular markers in combination with best practice clinical assessments. We chose to use LASSO regression modeling not only as it is known to perform well in these kinds of data sets but also because it is widely applied and often easier to interpret than other methodologies. Another consideration is that the patients studied here were prevalent cases. Moreover, all the patients recruited had a clinical diagnosis of idiopathic, heritable, or drug-induced PAH. It would be of interest to sample patients with other presentations of PAH and other cardiopulmonary diseases to better understand the clinical utility of our RNA signature.

In summary, we report a whole-blood RNA profile that distinguishes patients with PAH from healthy control subjects and reflects disease severity. Integration with genomic data suggests that SMAD5 is important in the development of PAH, prioritizing restoration of normal SMAD5 function as a target for therapeutic intervention.

## Supplementary Material

Supplements

Author disclosures
