# Supplementary material for: Whole-Blood RNA Profiles Associated with Pulmonary Arterial Hypertension and Clinical Outcome
Source: Am J Respir Crit Care Med. 2020 Aug 15;202(4):586–94. doi: 10.1164/rccm.202003-0510OC (PMC7427383; doi:10.1164/rccm.202003-0510OC)
Supplement: Supplements [file rccm.202003-0510OC.html]

Whole-Blood RNA Profiles Associated with Pulmonary Arterial Hypertension and Clinical Outcome | American Journal of Respiratory and Critical Care Medicine

- disclosures.pdf (609 KB)
- rhodes\_data\_supplement.pdf (1 MB)
- rhodes\_supplementary\_tables.xlsx (284 KB)
