# Supplementary material for: Whole-Blood RNA Profiles Associated with Pulmonary Arterial Hypertension and Clinical Outcome
Source: Am J Respir Crit Care Med. 2020 Aug 15;202(4):586–94. doi: 10.1164/rccm.202003-0510OC (PMC7427383; doi:10.1164/rccm.202003-0510OC)
Supplement: Supplements [file rccm.202003-0510OC_rhodes_data_supplement.pdf]

Online Appendix:

Whole blood RNA profiles associated with pulmonary arterial hypertension and clinical outcome

Short title: Whole blood RNAseq in PAH

Christopher J Rhodes 1* , Pablo Otero-Núñez 1*, John Wharton 1 , Emilia M Swietlik 2 , Sokratis Kariotis 3,4 , Lars Harbaum 1 , Mark J Dunning 5 , Jason M Elinoff 6 , Niamh Errington 3,4 , A. A. Roger Thompson 4 , James Iremonger 4 , J. Gerry Coghlan 7 , Paul Corris 8 , Luke S Howard 1 , David G Kiely 4, Colin Church 9 , Joanna Pepke-Zaba 10 , Mark Toshner 2 , Stephen J Wort 1 , Ankit A. Desai 11 , Marc Humbert 12 , Prof William C. Nichols 13 , Laura Southgate 14 , David-Alexandre Trégouët 15 , Richard C. Trembath 16 , Inga Prokopenko 17, Stefan Gräf 2,18 , Nicholas W Morrell 2 , Dennis Wang 3,4 , Allan Lawrie 6 , Martin R Wilkins 1

On behalf of the NIHR BioResource – Rare Diseases PAH Consortium and the UK National PAH Cohort Study Consortium; *these authors contributed equally

	Affiliation(s)	Country
1	National Heart and Lung Institute, Imperial College London	United Kingdom
2	Department of Medicine, University of Cambridge	United Kingdom
3	Sheffield Institute for Translational Neuroscience, University of Sheffield	United Kingdom
4	Department of Infection, Immunity & Cardiovascular Disease, University of Sheffield	United Kingdom
5	Sheffield Bioinformatics Core, The University of Sheffield	United Kingdom
6	Critical Care Medicine Department, National Institutes of Health Clinical Center	United States
7	University College London	United Kingdom
8	Newcastle University, Newcastle upon Tyne	United Kingdom
9	University of Glasgow	United Kingdom
10	Royal Papworth Hospital, Papworth	United Kingdom
11	Indiana University, Indianapolis IN	United States
12	Université Paris-Saclay, Faculté de Médecine; INSERM UMR_S 999; Assistance Publique - Hôpitaux de Paris, Department of Respiratory and Intensive Care Medicine, Hôpital Bicêtre, Le Kremlin-Bicêtre, France.	France
13	Division of Human Genetics, Cincinnati Children's Hospital Medical Center, Department of Pediatrics, University of Cincinnati College of Medicine	United States
14	Molecular and Clinical Sciences Research Institute, St George's University of London	United Kingdom
15	INSERM UMR_S 1219, Bordeaux Population Health research center, University of Bordeaux, Bordeaux	France
16	Division of Genetics and Molecular Medicine, King's College London	United Kingdom
17	Department of Clinical and Experimental Medicine, University of Surrey	United Kingdom
18	NIHR BioResource for Translational Research, Cambridge Biomedical Campus	United Kingdom

Correspondence to: Prof Martin R Wilkins, m.wilkins@imperial.ac.uk, Imperial College London, 2S4 Commonwealth Building, Hammersmith Campus, Du Cane Road, LONDON, W12 0NN, United Kingdom

Supplementary Figures

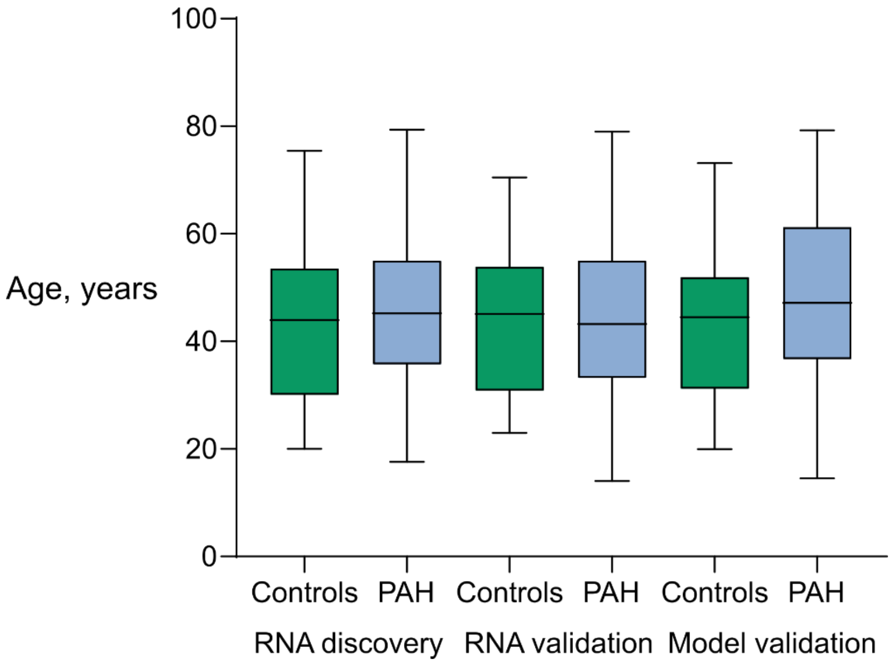

Figure E1 - Age in controls (green) and PAH patients (blue) in 3 analysis groups after randomisation of PAH patients and matching of controls.

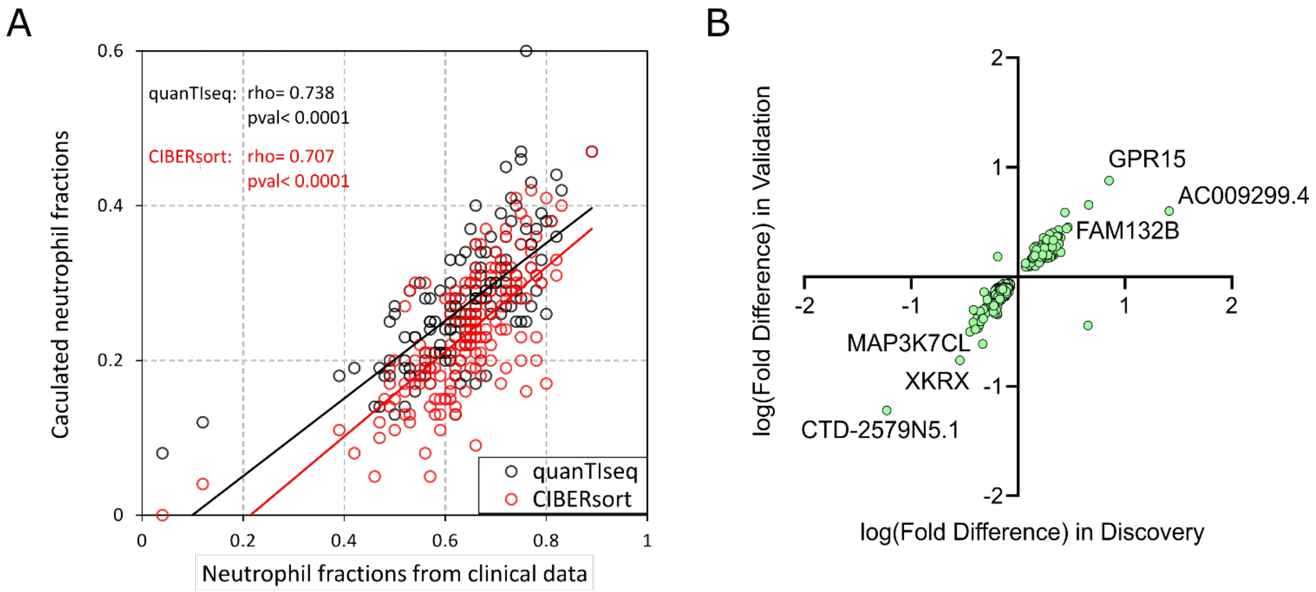

Figure E2 - A. Scatter plot of calculated neutrophil fractions by RNAseq deconvolution methods quanTIseq and CIBERsort in PAH patients where clinical measurements were also available. B. Scatter plot showing fold-differences in 509 RNAs reaching p<0.05 in both discovery and validation analyses. 507 directionally consistent genes were taken forward in the main analysis.

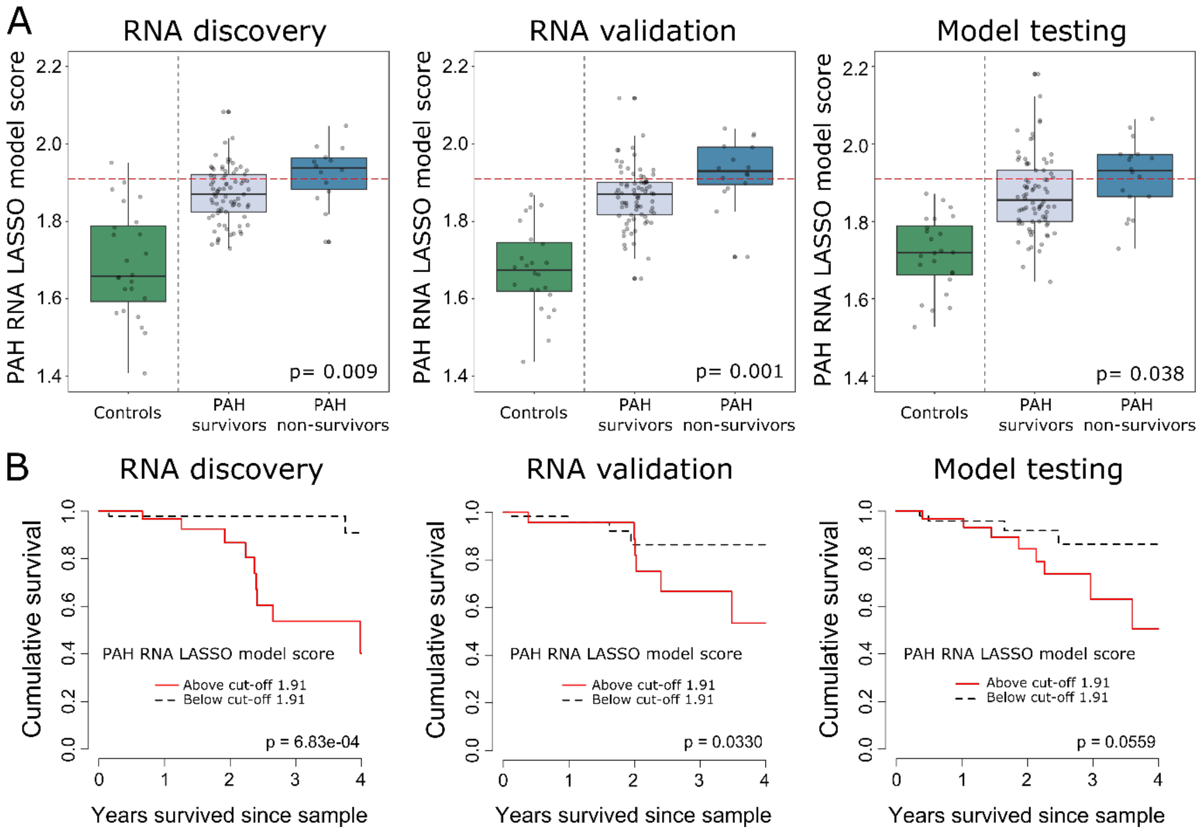

Figure E3 – Survival association of RNA model score sub-analysis in separate groups used for differential expression analysis. A. Boxplots showing distribution of model score between controls and patients who either survived or died during follow-up period. Red dashed line indicates prognostic cut-off derived by ROC in the main analysis of 1.91. B. Kaplan-Meier survival curves for PAH patients divided by RNA model score cut-off (red lines indicate higher than cut-off and dashed black lines not higher than cut-off).

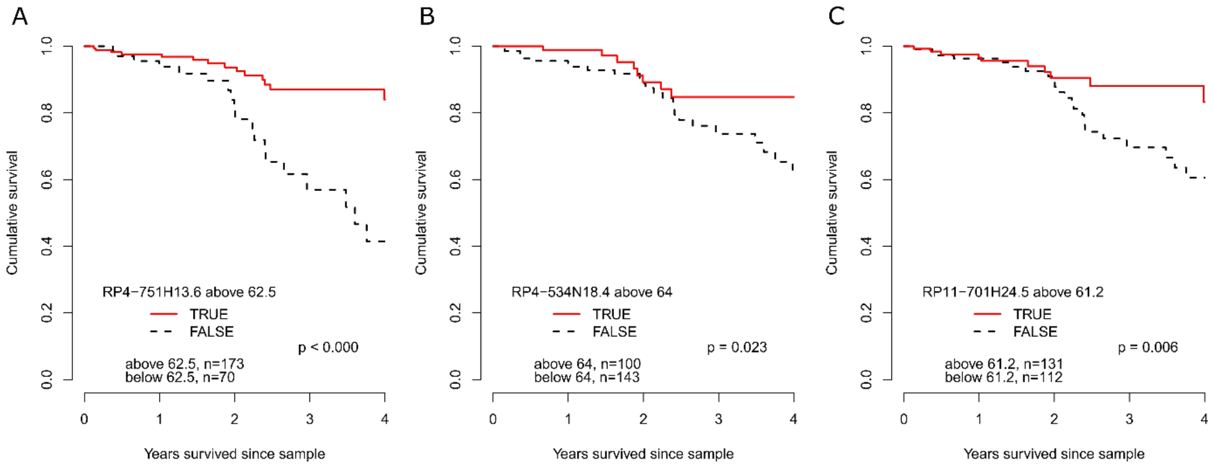

Figure E4: Kaplan-Meier survival plots for three diagnostic intronic long non-coding RNAs. A. RP4-751H13.6, also known as ATP6V0E2-AS1. B: RP4-534N18.4, also known as AL136115.3. C. RP11-701H24.5, also known as lnc-SNRPN-1:6.

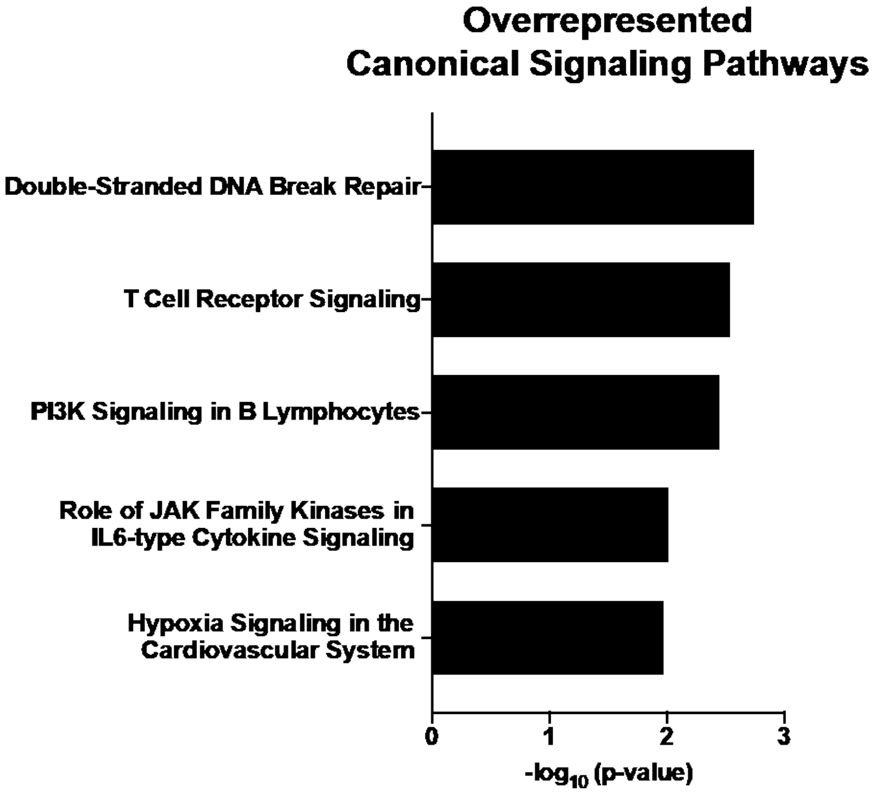

Figure E5: Selected top canonical pathways identified by IPA® *as overrepresented among the 507 differentially regulated PAH genes (uncorrected p≤0.01 for all). P-values are calculated by Fisher's exact test. Some annotations were modified for clarity. Supplementary Table 11 includes the full list of enriched canonical pathways.*

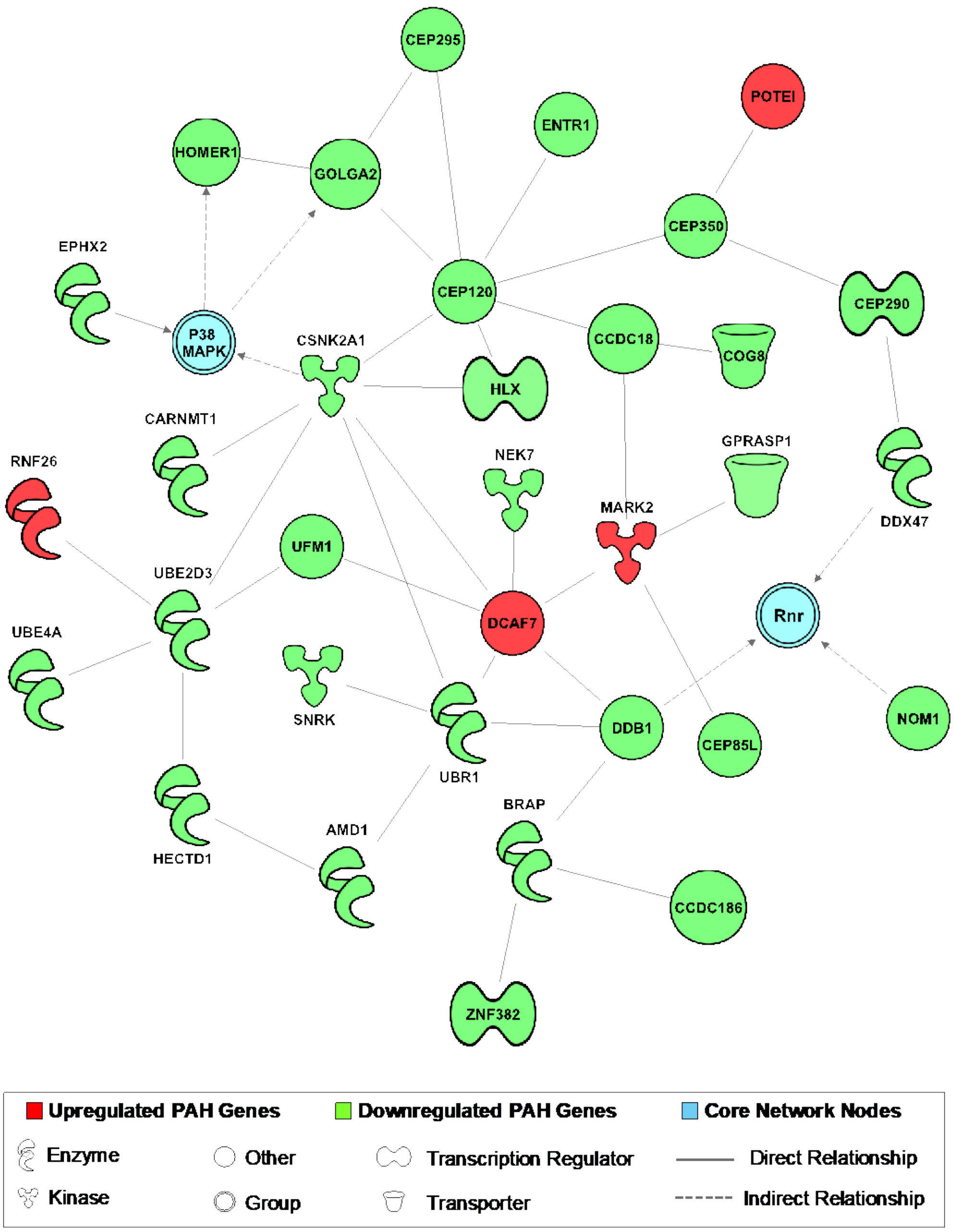

Figure E6: Top IPA® gene network identified from the 507 differentially regulated PAH genes.

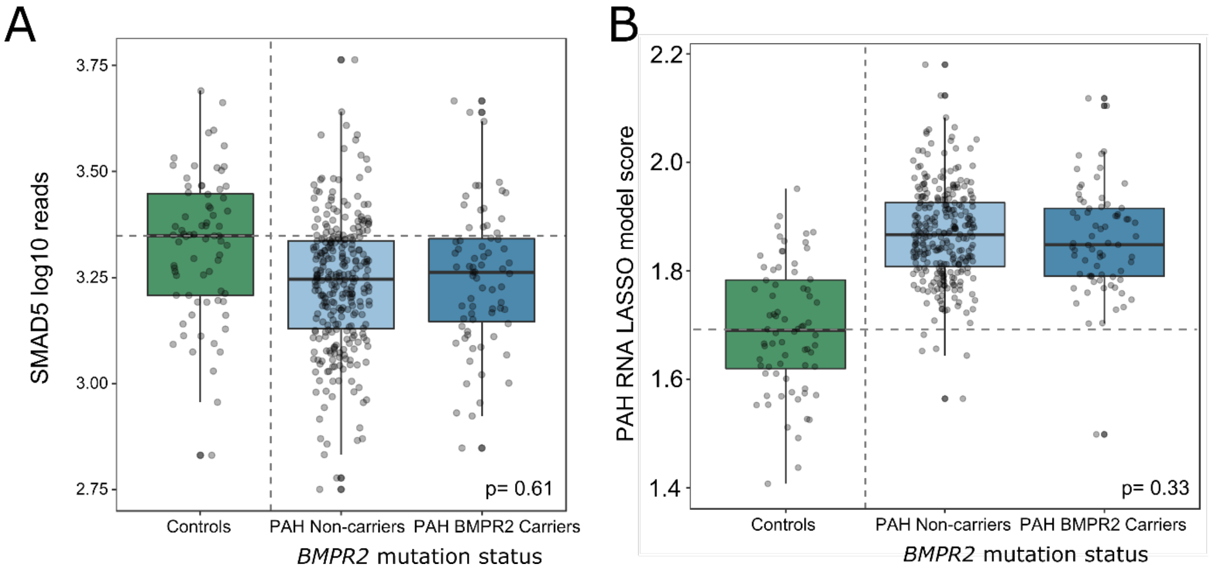

Figure E7 – A. SMAD5 levels in controls, and PAH patients divided by pathogenic BMPR2 variant carrier status B. RNA model score in controls, and PAH patients divided by pathogenic BMPR2 variant carrier status.

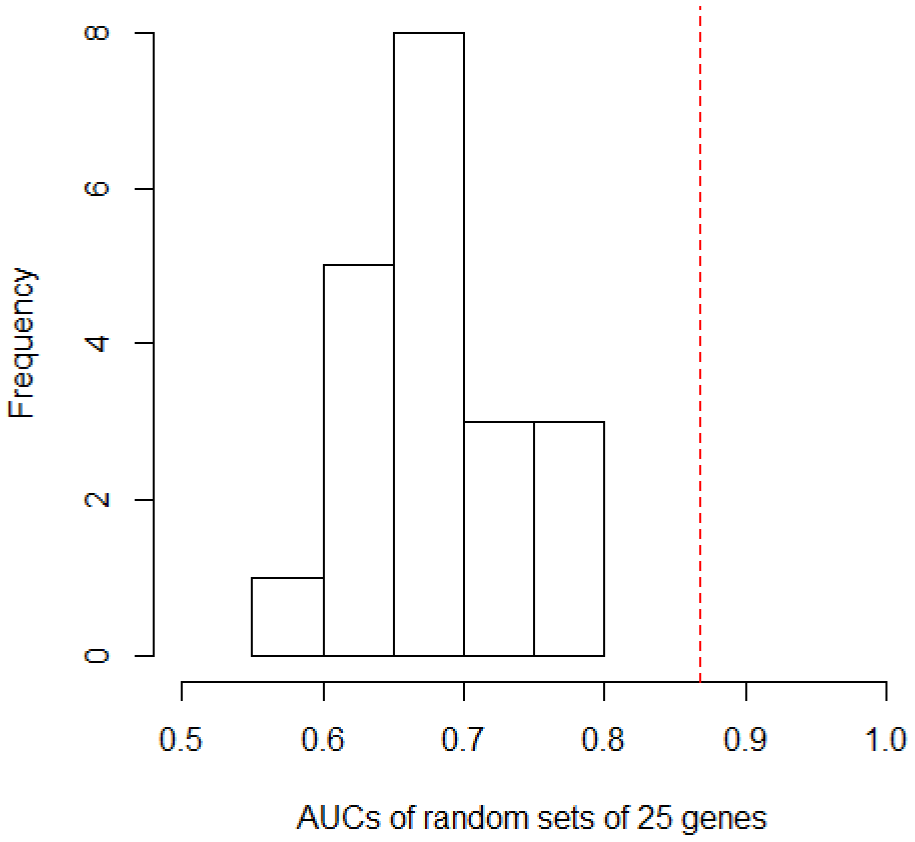

Figure E8 – Histogram of performance of models based on sets of 25 randomly selected genes from the 507 top hits in the model validation cohort (area under the curve). The red dashed line indicates the performance of the LASSO derived model in the main analysis for reference.

Supplementary methods

Study participants and sample analysis

Patients with idiopathic, heritable or drug-induced pulmonary arterial hypertension (referred to throughout as PAH) were recruited from expert centres across the UK as part of the PAH Cohort study (www.ipahcohort.com). In each case, diagnosis was confirmed by right heart catheterisation following international guidelines(1) which remained unchanged for the duration of this study; a mean pulmonary artery pressure >25mmHg at rest, with pulmonary vascular resistance >3 Wood units and mean pulmonary capillary wedge pressure <15 mmHg following exclusion of other PH aetiologies (left heart disease, lung disease, chronic thromboembolic PH or miscellaneous causes). Healthy volunteers were recruited at the same centres and samples processed using the same standard operating procedure at all sites. Acute vasoresponders were defined as patients who demonstrated a >=10 mmHg fall in mean pulmonary artery pressure (mPAP) to <= 40 mmHg without significant impact on cardiac output(1) in response to vasodilator therapy during right heart catheterisation. These patients go on to have improved clinical outcomes on calcium channel blocker vasodilator therapy. All individuals gave written, informed consent with local ethical committee approval.

359 PAH patients were randomised into 3 data analysis groups for RNA discovery (n=120), RNA validation (n=120) and model validation (n=119). Each of these 3 groups were then compared to an independent set of age- and sex-matched healthy volunteers without cardiac or respiratory disease as controls (n=24 in each set; Table 1 and Supplementary Figure 1).

Whole blood (3 ml) was collected in Tempus™ Blood RNA Tubes, which were stored at -80 ^{o}C until required. RNA was extracted using a Maxwell® 16 Tissue LEV Total RNA Purification Kit (Promega, Madison, WI, USA). All samples submitted for sequencing had a 260/230 ratio >1.5, a 260/280 ratio between 1.9-2.1 and an RNA integrity number (RIN)>7 as determined by Bioanalyser (Agilent, Santa Clara, CA, USA). Globin-Zero Gold rRNA Removal Kits (Illumina Inc, San Diego, CA) were used to remove ribosomal RNA contamination from whole blood RNA samples. 75 bp paired-end sequencing on a Hiseq4000 was performed on pooled multiplexed libraries of ~80 samples on 9 sequencing lanes. To minimise batch effects on analyses, each pool contained a randomised combination of controls and patients with similar age, sex and ethnicity profiles.

Genomics data including summary statistics for PAH association and genotypes of PAH patients recruited for this study were obtained from the UK NIHR BioResource whole genome sequencing rare diseases project and a published PAH genome-wide association study(8).

RNAseq validation through RT-qPCR of dysregulated genes

Reverse transcription (RT) and quantitative-PCR (q-PCR) were performed in RNA extracts from representative subjects of the model testing group. Assays were carried out on a selection of the most significantly dysregulated and biologically relevant genes, for example roles in the cell cycle/proliferation relevant to pulmonary vascular remodelling. Due to the variable levels observed in RNAseq assays of common 'housekeeping' genes that are often used as controls in qPCR analyses(33), a list of the most stable genes were defined as well expressed (0<log10 transcripts per million [tpm]<2) with the lowest variance (selected candidate genes var= 0.125-0.140). Following a

literature review of candidate genes, SPAST was selected due to its biological role as a microtubule binding and severing protein.

RT-PCR was performed on RNA samples using Multiscribe Reverse Transcriptase (Applied Biosystems, ThermoFisher Scientific, UK) and the resulting cDNA used for q-PCR using PowerSYBRgreen mastermix (Applied Biosystems, ThermoFisher Scientific, UK). Relative expression levels were calculated as 2^-(δCT [gene] – δCT [SPAST]). Expression levels were assessed in controls (randomly selected, lowest score to 55th percentile, n=11) and patients with both mid-range (40th – 70th percentile of all scores, n=18) and high RNA LASSO scores (90th percentile and above, n=18) to give dynamic range. These were also compared by Spearman's Rank Test with the expression values from the RNAseq assay to assess the correlation between methods.

RNAseq Data analysis

Fastq files (raw reads from RNAseq) were analysed using Salmon v0.9.1(9) and GENCODE release 28 to produce transcript abundance estimates which were converted to gene expression data using tximport in R with Rstudio(10). We used Salmon as it is the first transcriptome-wide quantifier to correct for fragment GC-content bias, which substantially improves the accuracy of abundance estimates and the sensitivity of subsequent differential expression analysis(9). Well detected genes (n=25966) with 2 or more reads in at least 95% of control or patient samples were considered for downstream analyses.

Principal components analysis using overall gene expression data for quality control identified one significant outlier which was excluded from further analyses. No clustering by RNA extraction or sequencing batch was observed. The first 3 principal components were used to correct for remaining data structure in downstream analyses.

Deconvolution of white blood cell fractions from RNAseq data

RNAseq analysis of tissues with mixed cell types such as blood can be affected significantly by the cell composition of each sample. Two different computational approaches, CIBERsort(11) and quanTIseq(12), were used to predict white blood cell (WBC) profiles based on RNA signatures obtained from the preliminary RNAseq assay (n= 25,966 genes). Estimated WBC fractions from both computational approaches were used as covariates to generate a logistic regression model for PAH based on WBC fractions. A backward stepwise process was followed to generate the best performing model based on Akaike information criterion (AIC). Predicted profiles correlated (rho= 0.44-0.73) with clinical white cell fractions available in a subset of patients (Supplementary Figure 2. Each approach revealed WBC fractions that were different between PAH and control individuals (Supplementary Table 1). Logistic regression analysis identified the fractions predicted by CIBERsort (naïve CD4+ T cells, memory B cells, resting mast cells, resting dendritic cells) and quanTIseq (regulatory T cell/Treg, CD4+ T cells) that independently differentiated PAH patients from controls (Supplementary Table 2). These cell subtypes driving this model were included as covariates in secondary differential gene expression analyses.

Differential expression analysis was performed using edgeR v3.22.5(13) correcting for principal components. Differentially expressed genes were defined in analyses both with and without WBC fractions as covariates in distinct discovery and validation sample sets. These sets were then

combined and only significant genes ($p<0.05$) and directionally consistent in the initial analyses and meeting false discovery rate multiple test corrections (based on all detected genes, $\alpha=0.1$) in the combined analysis were taken forward. 507 genes meeting these criteria were considered to generate a model to distinguish PAH from controls. Subset selection of RNAs which best distinguish PAH in combination was performed by least absolute shrinkage and selection operator (LASSO) regression analysis, using the glmnet v2.0-18 package from CRAN[8], with k-fold cross-validation (k=10) selecting the largest value of lambda such that error is within 1 standard error of the minimum. This produces an RNA score from a linear weighted combination of the mRNAs identified by the LASSO analysis. Receiver operating characteristic (ROC) analysis was performed using the pROC v1.14.0 package from Bioconductor(14). Optimal cut-offs were determined by the maximal Youden index (sensitivity + specificity-1).

Association testing

Survival curves from date of diagnostic right heart catheterisation were constructed using Kaplan-Meier estimates with left-truncation for date of sampling for this study to correct for survival bias. Differences in survival estimates were assessed by log rank test. RNA scores were also compared across disease severity markers 6-minute walk test (Spearman's rank), WHO functional class (Kruskal-Wallis ANOVA) and by cardiac biomarkers - circulating BNP or NT-proBNP as available, divided by presence of low, intermediate or high levels of cardiac biomarkers BNP <50 pg/ml, 50-300 pg/ml or >300pg/ml, respectively or NT-proBNP <300 pg/ml, 300-1400 pg/ml or >1400 pg/ml, respectively, as per European guidelines for PAH assessment. for risk assessment(1).

Functional annotation and enrichment of the genes associated with PAH was performed using DAVID (david.ncifcrf.gov) and Ingenuity Pathway Analysis (IPA®).

Mendelian randomisation analysis using all independent genome-wide significant whole blood expression quantitative trait loci (eQTLs) from two published studies(15, 16) and PAH association from our published GWAS(8) was performed using the TwoSampleMR package (17). Expression quantitative trait loci (eQTL) in cis to the target gene (cis-window of transcription start site ± 500 kb) were clumped with a linkage disequilibrium (LD) threshold of 0.01.

Supplementary Results

Differential expression analysis was performed to investigate if gene expression was associated with exposure to the main PAH therapies (PDE5 inhibitors, endothelin receptor antagonists and prostanoids), using the same discovery and validation groups used in the main analysis, correcting for 3 principal components and white blood cell profiles. Four, seven and fifty genes, respectively, were differentially expressed in patients exposed to PDE5 inhibitors, ERAs or prostanoids prior to sampling for this study (Supplementary Table 14). None of the 507 genes associated with PAH were associated with drug treatments.

Random sets of 25 genes (20 sets, without repeating genes) were selected from the 507 top hits to construct logistic regression models to predict PAH in the model derivation groups, and performance

was tested in the model validation group, as per the LASSO model presented in the main paper. The mean AUC was 0.679 vs 0.868 for the LASSO derived model presented, ranging from 0.595 – 0.779, meaning the LASSO-derived model was better than all 20 models based on randomly selected PAH-associated genes (Supplementary Figure 8).
